# FAK inhibition with small molecule inhibitor Y15 decreases viability, clonogenicity, and cell attachment in thyroid cancer cell lines and synergizes with targeted therapeutics

**DOI:** 10.18632/oncotarget.2381

**Published:** 2014-08-25

**Authors:** Shalana O'Brien, Vita M. Golubovskaya, Jeffrey Conroy, Song Liu, Dan Wang, Biao Liu, William G. Cance

**Affiliations:** ^1^ Department of Surgical Oncology; Roswell Park Cancer Institute, Buffalo, NY; ^2^ University at Buffalo/State University of New York, Buffalo, NY; ^3^ Genomics Shared Resource, Center for Personalized Medicine, Roswell Park Cancer Institute, Buffalo, NY; ^4^ Bioinformatics Core, Biostatistics, Roswell Park Cancer Institute, Buffalo, NY

## Abstract

Focal adhesion kinase (FAK) is up-regulated in thyroid cancer and small molecule FAK scaffolding inhibitor, Y15, was shown to decrease cancer growth in vitro and in vivo. We sought to test the effectiveness of Y15 in thyroid cancer cell lines, profile gene expression with Y15 compared with clinical trial FAK inhibitor PF-04554878, and use Y15 in novel drug combinations. Cell viability was decreased in a dose dependent manner in four thyroid cancer cell lines with Y15 and with higher doses in PF-04554878. Y397 FAK and total FAK were decreased with Y15 and decreased less with PF-04554878. Detachment and necrosis were increased in a dose-dependent manner in all cell lines with Y15. Clonogenicity was decreased in a dose-dependent manner for both Y15 and PF-04554878. We compared gene profiles between papillary thyroid cell lines, TPC1, BCPAP and K1, and 380, 109, and 74 genes were significantly >2-fold changed with Y15 treatment, respectively. Common up-regulated genes were involved in apoptosis, cell cycle, transcription and heat shock; down-regulated genes were involved in cell cycle, cell-to-cell interactions, and cancer stem cell markers. We also compared gene profiles of TT cells treated with Y15 versus PF-04554878. Y15 caused 144 genes to change over 4 fold and PF-04554878 caused 208 gene changes >4-fold (p<0.05). Among genes changed 4 fold, 11 were shared between the treatments, including those involved in metabolism, cell cycle, migration and transcription. Y15 demonstrated synergy with PF-04554878 in TT cells and also synergy with Cabozantinib, Sorafenib, Pazopanib, and strong synergy with Sunitinib in resistant K1 cells. This report revealed the biological effect of Y15 inhibitor, detected the unique and common gene signature profiles in response to Y15 in 4 different thyroid cancer cell lines, demonstrated differential response changes with Y15 and PF-04554878 treatment, and showed the synergy of Y15 with PF-04554878, Cabozantinib, Sorafenib, Pazopanib, and Sunitinib.

## INTRODUCTION

Over 60,000 new cases of thyroid cancer are diagnosed each year in the United States, comprising over 95% of endocrine malignancies. It is the fastest increasing cancer, with rates rising 5-7% every year [1] in the US and also increasing worldwide. Most of the thyroid cancer cases diagnosed are papillary or follicular thyroid carcinomas, which are derived from thyroid follicular epithelial cells. About 5% of cases are medullary thyroid cancer, derived from neuroendocrine parafollicular cells that secrete calcitonin. Approximately 1% of thyroid cancers are anaplastic, a dedifferentiated tumor, or tumors of non-thyroid origin, such as sarcoma and lymphoma. Papillary thyroid cancer is usually treated with total thyroidectomy, with or without lymphadenectomy. If the tumor takes up iodine, radioactive iodine can be used to destroy any remaining tumor, but there are no chemotherapeutic or directed therapies used regularly. Early stages of medullary thyroid cancer are treated similarly, but due to its aggressiveness, aggressive treatment with lymphadenectomy and radiation is done more frequently [2]. Additionally, advanced medullary cancer can now be treated with adjuvant targeted therapies: recently FDA approved tyrosine kinase inhibitors, Vandetanib and Cabozantinib [3]. There are also drugs undergoing clinical trials to treat thyroid cancer: Sorafenib, Pazopanib, and Sunitinib. All three of these drugs inhibit VEGFR-1, -2, -3, and PDGFR-β. In addition, Sorafenib also inhibits Raf-1 and B-Raf; Pazopanib also inhibits FGFR-1, -3, c-kit and c-fms; and Sunitinib also inhibits RET. These are similar to the approved drugs: Vandetanib which targets RET, VEGFR, and EGFR; and Cabozantinib which also inhibits RET and VEGFR2 and additionally inhibits c-met [4]. While thyroid cancer can be curable with resection of low stage tumors, especially papillary thyroid cancer, new treatments are needed for advanced differentiated cancers with radioiodine resistance.

In order to overcome the current radioiodine resistance within thyroid cancer, identifying and targeting other proteins of interest may work in tandem to effectively treat thyroid cancer. Focal Adhesion Kinase (FAK) is one of these targets. FAK is expressed in all cells at a low basal level, however it is significantly overexpressed in a majority of solid tumors, including papillary carcinomas, with even higher levels of expression in metastatic tumors [5]. The focal adhesion complexes where FAK resides not only helps tether the cell to the extracellular matrix, but also is a hub for signal transduction, mediated by FAK. FAK's autophosphorylation site at Y397 allows for the binding of Src, PI3 kinase, Grb-7, Shc, and other SH2 domain containing proteins. The binding of Src to the phosphorylated Y397 leads to downstream signaling and mediates the further phosphorylation of FAK [6]. Activation of FAK results in increased cell survival, motility, and proliferation, leading to angiogenesis, metastasis, and invasion of tumors. FAK is therefore identified as a promising cancer drug target.

One FAK inhibitor, PF-04554878, is in a phase I clinical trial for ovarian cancer (clinical trial #NCT01778803) [7]. Recently a FAK autophosphorylation inhibitor was identified: 1,2,4,5-Benzenetetraamine tetrahyrdrochloride (called Y15) [8]. Y15 treatment *in vitro* resulted in decreased cell viability, increased detachment, and increased apoptosis in colon cancer cells [9], breast cancer cells, and melanoma [8]. Y15 was also tested *in vivo* using colon cancer cell lines and caused decreased tumor growth. It also demonstrated an enhancement in tumor growth inhibition when combined with 5-FU and oxaliplatin [9]. The mechanism by which Y15 affects thyroid cells is still unknown although other tyrosine kinase inhibitors have been shown to be effective. In this report, we compared the effect of Y15 in different thyroid cancer cell lines, compared gene expression in three papillary thyroid cell lines in response to Y15 and also compared the effect of Y15 and PF-04554878 inhibitors in a medullary cell line, TT.

## MATERIALS AND METHODS

### Cell lines and culture conditions

The thyroid cancer cell lines: TPC1, TT and BCPAP were maintained according to protocol in RPMI plus 10% fetal bovine serum (FBS) and 1% penicillin/streptomycin (37 °C, in 5% CO2). K1 was maintained in a 2:1:1 ratio of Dulbecco's modified Eagle, Ham's F-12, and MCDB 105 mediums plus 2mM Glutamine, %10 FBS and 1% penicillin/streptomycin.

### Antibodies

Monoclonal (clone 4.47) anti-FAK antibody (Millipore) and polyclonal anti-phospho-Tyr397 FAK antibody (Enzo) were used to detect total and phosphorylated FAK. Monoclonal GAPDH antibody was used for a loading control (Invitrogen). Secondary mouse (from sheep) and rabbit (from donkey) horseradish peroxidase linked IgG (GE Healthcare UK Unlimited) were used for the secondary antibodies.

### Small molecule inhibitors

1,2,4,5-Benzenetetraamine tetrahyrdrochloride (Y15) is an allosteric FAK inhibitor and was ordered from Sigma. Y15 was dissolved in 1× PBS at a concentration of 25 mM and stored at −20 °C. Pazopanib, Sunitinib, and Sorafenib were ordered from LC Laboratories and reconstituted in DMSO at a concentration of 25mM. Cabozantinib was ordered from Selleck Chem and solubilized in DMSO at 25mM. PF-04554878 was obtained from Adooq Bioscience and dissolved in DMSO to concentration of 25mM.

### Western blot analysis

Cells were washed with 1× PBS and collected by trypsinization. Collected cells were incubated on ice in lysis buffer [50 mM TRIS-HCl (pH 7.5), 150 mM NaCl, 1% Triton X-100, 0.5% sodium deoxycholate, 0.1% SDS, 10% glycerol] with protease inhibitors (10 μg/mL leupeptin, 10 μg/mL PMSF, 1 μg/mL aprotinin, 1 mM sodium vanadate and 5 mM sodium fluoride) for 30 min. Lysed cells were spun in a centrifuge to collect protein samples which were stored at −80 °C. Protein samples (15 μg) were separated using electrophoresis and transferred onto an Immobilon-P PVDF membrane (Millipore), then incubated for one hour in 1% BSA/0.01% Tween-20 in PBS. The membranes were washed three times with 0.01% Tween-20 in PBS and then were incubated with primary antibody for one hour. The membranes were again washed three times with 0.01% Tween-20 in PBS before and after being incubated in secondary antibody for one hour. The membranes were then developed with Western Lightning Plus-ECL Enhanced Luminol Reagent Plus (PerkinElmer). Photoshop and Illustrator were used to prepare images from scanned films.

### Viability

Cells were seeded onto 96-well plates (10,000 cells per well in 100 μL of medium plus 10% FBS and 1% penicillin/streptomycin). Twenty-four hours following inhibitor treatment, 20 μL of Cell Titer 96 Aqueous One Solution Cell Proliferation Assay (Promega) was added to each well. After two hours of incubation with reagent and the plate was read with the Gen 5 1.07 microplate reader at 490 nm. Each sample was analyzed in triplicate.

### Clonogenicity

Two 6-well plates were seeded with TPC1 and K1 cells at a concentration of 500 cells per well in 2 mL of media. BCPAP cells were plated at 1500 cells and 2mL of media per well. After overnight incubation (37 °C, 5% CO2), each well was treated with a different Y15 doses and left to incubate. After 14 days the media was aspirated from each well and a solution of 1% methylene blue and 50% methanol was added. The cells were incubated in this solution for 30 minutes to fixate and stain them. The colony number in each well was counted in duplicate plates.

### Detachment

Six-well plates were seeded with TPC1, K1, BCPAP and TT cells in respective media at a concentration of 2 × 10^6 and allowed to incubate overnight to attach to the plate. After 24 hours, each well was treated with a specific Y15 dose. After an additional 24 hours, the media from each well was collected and the number of unattached cells in each well was counted three times with a hemocytometer. The attached cells were collected with trypsinization and counted in the same manner. For each treatment condition the average percent of detached cells was calculated by dividing the number of detached cells by the number of total (attached plus detached) cells.

### Apoptosis

Apoptosis assay was done with PE Annexin V Apoptosis Detection Kit (BD) to identify apoptotic nuclei. One hundred millimeter plates were seeded with TPC1, K1, BCPAP and TT cells at a concentration of 1 × 10^6 cells in normal growth media. After overnight incubation, plates were treated with one of several doses of Y15 or left untreated. After another 24 h of incubation, cells were collected with trypsin, and following centrifugation, were washed with 1× PBS. Again, cells were centrifuged, PBS was removed and cells were resuspended in binding buffer and filtered through BD mesh tubes. Annexin V and 7-AAD were added to the solution, vortexed and incubated for 15 minutes in the dark. Binding buffer was added and the cells were analyzed by flow cytometry within 1 hour.

### IC50 Curve Calculation

GraphPad Prism 6 software was used to graph cell viability data. Raw data was matched to nonlinear regression dynamic fitting curves for dose inhibition. Statistical analyses were done using this program and inhibitory concentration at 50% were calculated using 3 or 4 parameter variable slope log inhibitor response curves.

### Microarray Assay

Microarray assay was used to detect the changes in gene expression between cell lines and with Y15 and PF-04554878 treatment. One hundred millimeter plates were seeded with 1×10^6^ cells of TT cells. After incubating 24 hours to allow cell attachment the plates were treated with 10 μM Y15 and 10 μM PF-04554878 in duplicate and two plates were left untreated. The plates were allowed to incubate for 24 hours and then the cells were collected. The cells were submitted for gene expression profiling to the gene microarray facility. HumanRef 8 whole genome gene expression array and direct hybridization assay (Illumina) was used to derive cDNA from 500ng total RNA. The Ambion Illumina Total Prep RNA Amplification Kit (Ambion) was used to make biotin-labeled cRNA via *in vitro* transcription of the cDNA. Illumina HumanRef-8 v3 Bead Chips were made through labeling and hybridizing probes at 58 ° C overnight and then the fluorescence of each probe was measured; the Bead Chips were analyzed with the Illumina Bead Array. The data were entered into the NCBI Database with GEO accession numbers GSE55619 and GSE55603.

### Bioinformatics and Statistical Analysis

The microarray data were analyzed by the R-based Bioconductor package. The expression intensity was converted to a log2 scale and then the Quantile normalization algorithm was used to normalize; according to the lumi model. Next, the limma program was used to calculate the degree of expression for each gene. A significant difference in gene expression was represented by p-value <0.05 and fold change at least 1.2.

### Statistics

Student's t-test in Excel was used to calculate p-values for determining statistical significance between values for MTS, detachment, and apoptosis assays. Threshold for p-value was set to <0.05.

## RESULTS

### Thyroid cancer cells expressed variable levels of total and phosphorylated FAK, which decreased with Y15 treatment

**Figure 1:**
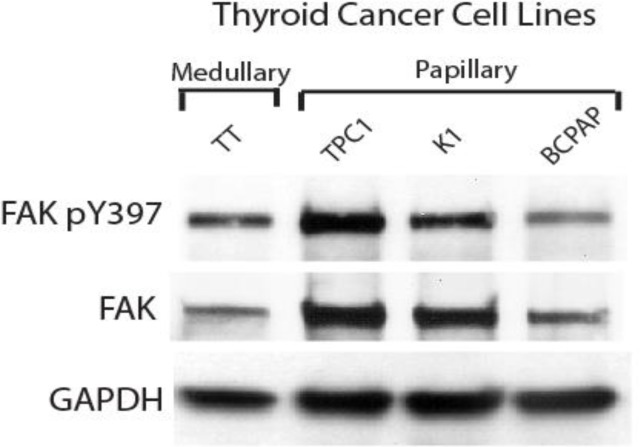
Expression of pY397 FAK and FAK in thyroid cancer cell lines Western blotting demonstrated expression of phosphorylated and total FAK between untreated medullary and papillary thyroid cancer cell lines.

At first, we compared the level of expression of FAK in untreated thyroid cancer cell lines (Figure 1). The medullary cell line, TT, had comparatively low levels of total FAK but a high level of phosphorylated FAK at tyrosine 397 (pY397). In the papillary cell lines, TPC1 had high levels of total FAK, but even higher expression of pY397 FAK. K1, another papillary cell line, had intermediate expression of both FAK and pY397 FAK and BCPAP, also a papillary cell line, had lower levels of total FAK and intermediate expression of Y397, which was the lowest expression of Y397 among all the cell lines (Figure 1). Thus, all thyroid cell lines expressed variable (intermediate and high) levels of Y397-FAK and FAK.

**Figure 2:**
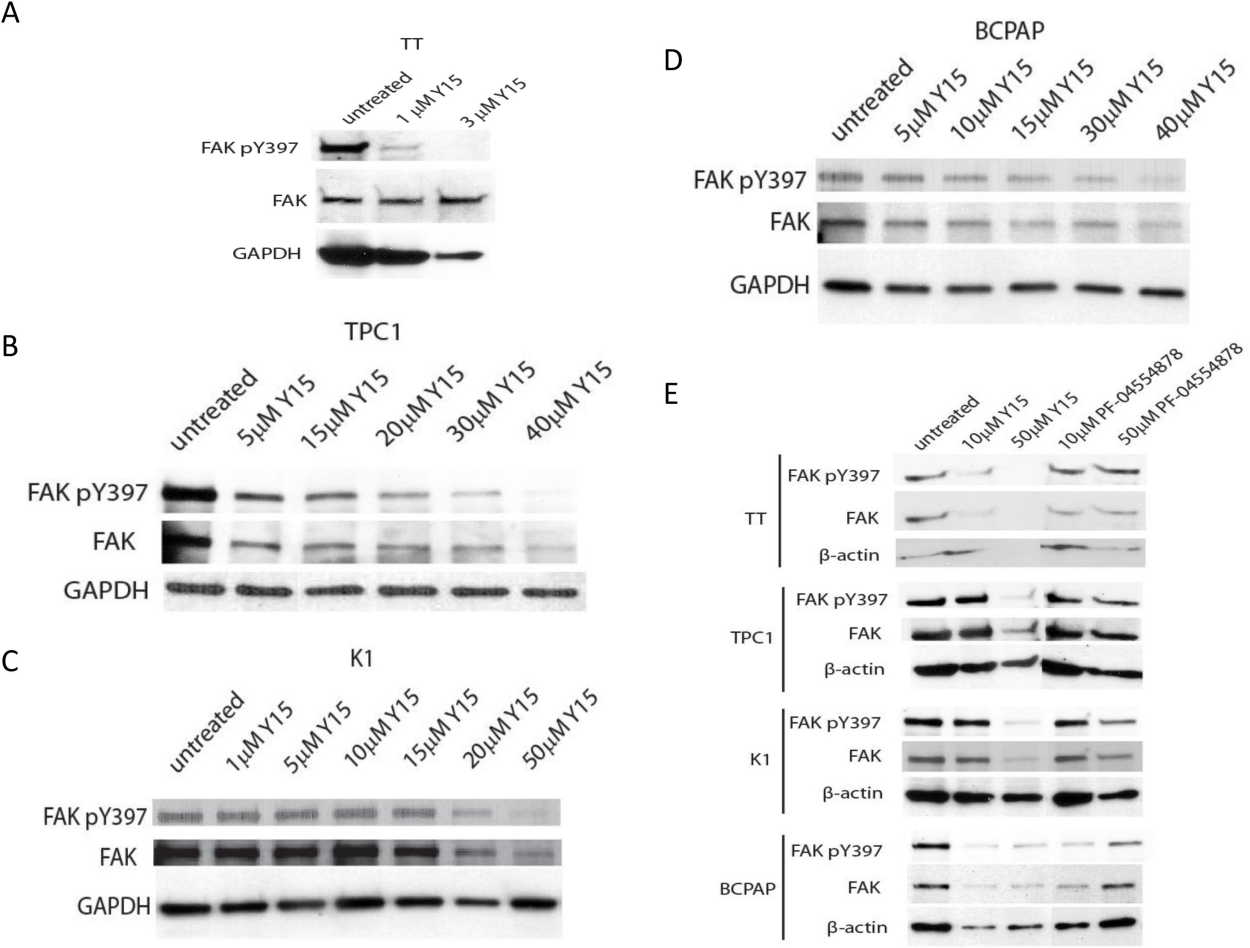
Y15 decreased pY397 FAK and FAK expression in a dose-dependent manner in thyroid cancer cell lines Western blot demonstrated Y15 decreased expression of phosphorylated and total FAK in four thyroid cancer cell lines in a dose-dependent manner. **(A)** In medullary cell line TT Y397-FAK expression effectively decreased at 3 μM with Y15 treatment. In papillary cell lines expression of Y397-FAK and FAK is also decreased by Y15: in TPC1 at 30 μM **(B)**, in K1 at 50 μM **(C)**, and in BCPAP at 40 μM **(D)**. **(E)** PF-04554878 decreased less pY397 FAK and FAK expression than Y15. PF-04554878 decreased Y397 FAK/FAK expression in TT, TPC1, K1, or BCPAP cell lines less than Y15 inhibitor.

In order to investigate the effect of Y15 on autophosphorylated and total FAK expression Western blotting was performed with Y397-FAK and FAK antibodies in TPC1, BCPAP, K1 and TT cell lines. In each cell line Y15 inhibited pY397 and total FAK expression in a dose-dependent manner. TT was the most sensitive cell line with effective inhibition of pY397 expression by 3 μM Y15 (Figure 2A). TPC1 cells had inhibition of phosphorylated FAK and total FAK expression at 30 μM Y15 (Figure 2B). K1 had Y397-FAK and FAK inhibition at 50 μM (Figure 2C) and BCPAP had inhibition of pY397 and FAK expression levels at 40 μM Y15 (Figure 2D). Thus, Y15 decreased pY397 and total FAK levels in a dose-dependent manner, most in the medullary thyroid cancer cell line and to a lesser extent in the papillary thyroid cancer cell lines.

We also tested the effect of the Pfizer inhibitor PF-04554878 (now VS-6063) on thyroid cancer cell lines to compare with Y15 inhibitor. In TT, TPC1, K1, and BCPAP cells PF04554878 did not change the expression of Y397 FAK and FAK as much as Y15 (Figure 2E). Thus, PF-04554878 had less effect on Y397 FAK and FAK expression compared to Y15 inhibitor.

### Y15 inhibited cell viability in a dose-dependent manner in all thyroid cancer cell lines

**Figure 3:**
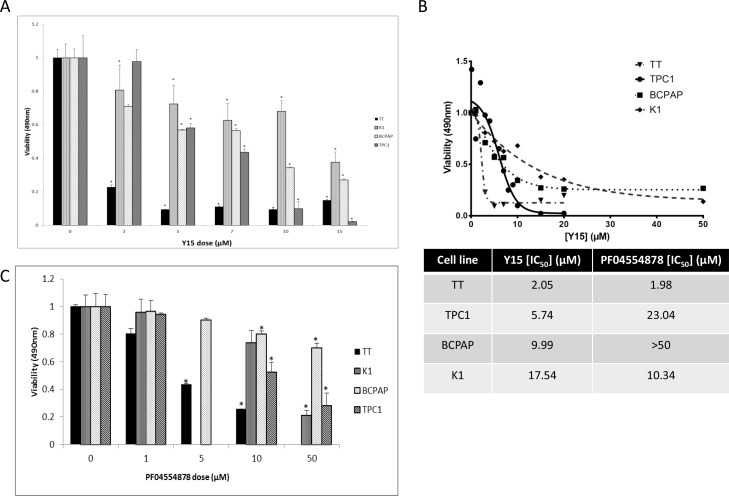
Y15 and PF-04554878 decreased cell viability in a dose-dependent manner in thyroid cancer cell lines **(A)** MTS assay demonstrated Y15 inhibited cell viability in a dose-dependent manner in four thyroid cancer cell lines. **(B)** Upper panel: The inhibitor response curves demonstrated cell line response to Y15. Lower panel: IC50 values calculated using GraphPad Prism from each Y15 dose response curve as described in Materials and Methods. The TT cell line had a greater response than TPC1, which had a greater response than BCPAP, which had a greater response than K1. Right panel: IC_50_ values calculated for PF-04554878 inhibitor compared to Y15 calculated from Figure 3C. **(C)** MTS assay demonstrated PF-04554878 inhibited cell viability in four thyroid cancer cell lines. *p<0.05 by Student's t-test.

Next we evaluated the effect of FAK inhibitor, Y15, on cell viability. MTS assay was completed using a range of Y15 doses on all cell lines (TT, K1, BCPAP, and TPC1, respectively).Y15 inhibited cell viability in a dose-dependent manner across all thyroid cell lines evaluated (Figure 3A). The medullary cell line, TT, was most sensitive, with significant inhibition of viability after 3 μM Y15 treatment, compared to untreated cells (p<0.05). Papillary cell line, K1, also demonstrated statistically significant inhibition of viability when treated with Y15 above 3 μM dose, but only showed small decreases with higher doses. BCPAP and TPC1 cell viabilities were significantly inhibited with Y15 treatment at the 5 and 6 μM dose, respectively. In conclusion, Y15 inhibited viability in all four thyroid cancer cell lines in a dose-dependent manner.

IC_50_ values of each cell line were calculated as described in Materials and Methods and are shown in Figure 3B (Figure 3B). IC_50_ values allow determination of sensitivity by cell viability of these cells to Y15: in decreasing sensitivity order TT, TPC1, BCPAP, and K1 that was consistent with decreased Y397-FAK levels by Y15 in these cells.

In addition we tested cell viability with PF-04554878 in MTS assays with the same four cell lines (Figure 3C). PF-04554878 decreased cell viability in a dose-dependent manner in all cell lines, but in most cell lines at higher doses compared with Y15 treatment. IC_50_ concentrations were also calculated for PF-04554878. IC_50_ values for TT was 1.98, TPC1 was 23.04, K1 was 10.34 and BCPAP was >50 μM (Figure 3B). TPC1 and BCPAP cell lines were more resistant to PF-04554878 than Y15 and decreased Y397-FAK less with PF-04554878 than Y15 (Figure 2E). Thus, TPC1 and BCPAP thyroid cell lines are more sensitive to Y15 than to PF-04554878 inhibitor, K1 cells are more sensitive to PF-04554878, and TT cells have similar sensitivities to both inhibitor suggesting different mechanisms of both inhibitors.

### Y15 increased detachment in each cell line in a dose-dependent manner

**Figure 4:**
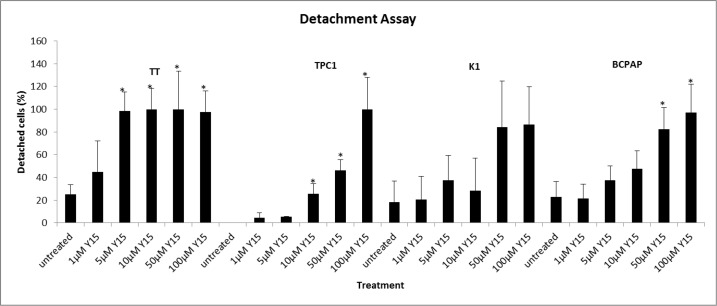
Y15 increased detachment in a dose-dependent manner in thyroid cancer cell lines Detachment assay demonstrated that Y15 increased detachment in TT, TPC1, K1, and BCPAP thyroid cancer cells in a dose-dependent manner. *p<0.05 by Student's t-test.

In order to understand the mechanism of Y15 and its role on FAK function in thyroid cells, the detachment assays were completed with Y15 treatment. Each cell line was treated with an increasing concentration of Y15 and detachment of these cells was evaluated. All cell lines demonstrated increased detachment with Y15 treatment in a dose-dependent manner (Figure 4). The medullary cell line, TT, was most sensitive to Y15 treatment with >90% detachment at over 5 μM Y15, which is consistent with low IC_50_ concentration and Y397-FAK in response to Y15. The papillary cell lines; BCPAP, K1 and TPC1; had >90% detachment at higher doses: 50-100 μM. In summary, Y15 caused effective dose-dependent detachment in all thyroid cancer cell lines.

### Y15 decreased clonogenicity in papillary thyroid cancer cell lines in a dose-dependent manner

**Figure 5:**
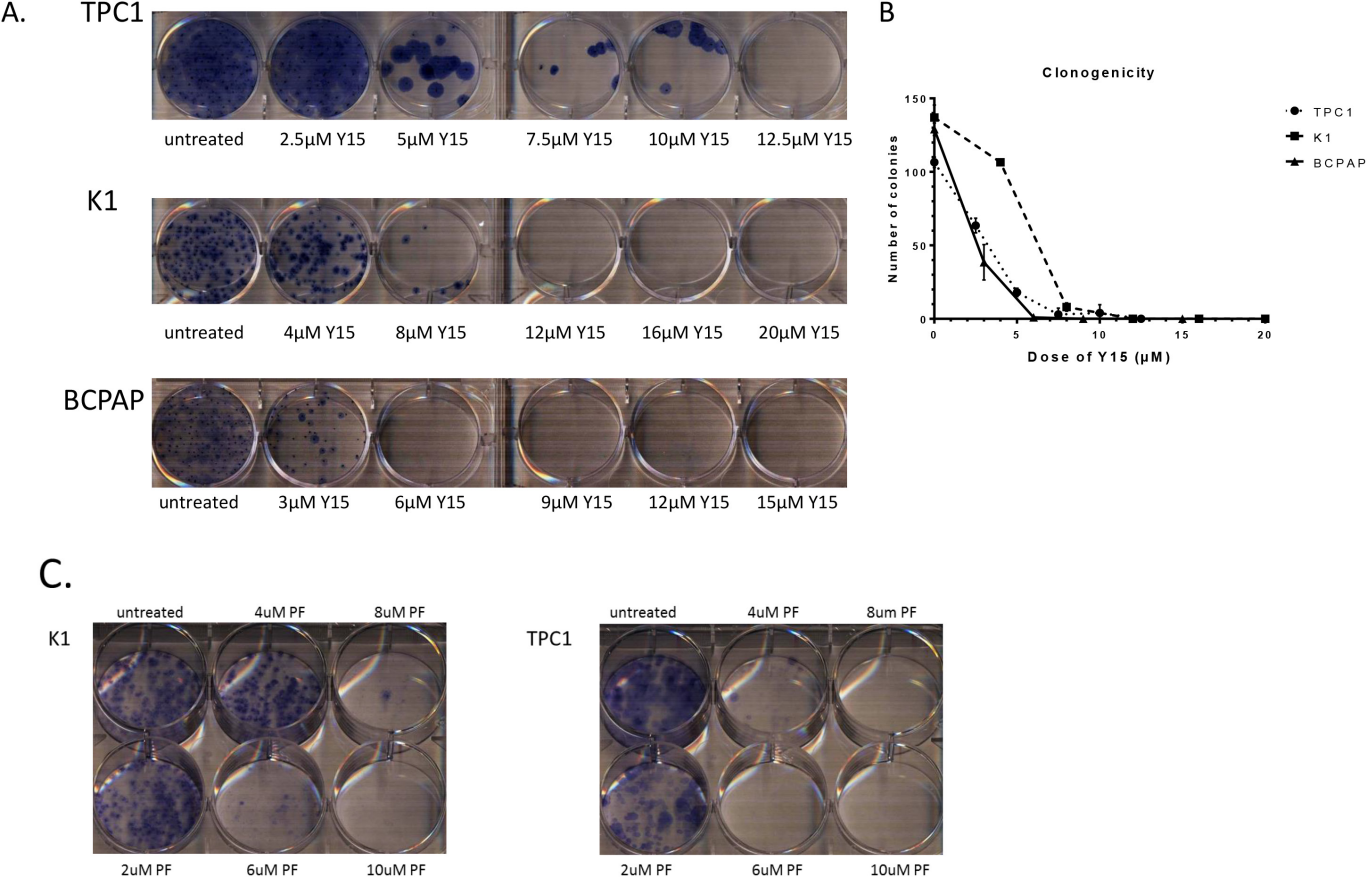
Y15 and PF-04554878 decreased clonogenicity in a dose-dependent manner in papillary thyroid cancer cell lines **(A)** Clonogenicity assay demonstrated colony growth decreased with Y15 treatment in papillary thyroid cancer TPC1, K1, and BCPAP cells. **(B)** Colony counts from the clonogenicity assay with TPC1, K1 and BCPAP cells demonstrates complete inhibition of colony formation with Y15 at 12.5 μM in TPC1, 12 μM in K1, and 6 μM in BCPAP. **(C)** Colony growth is inhibited by PF-04554878 in a dose-dependent manner in papillary thyroid cancer K1 and TPC1 cells. *p<0.05 by Student's t-test.

To further evaluate the effect of FAK inhibition on the papillary thyroid cell lines (K1, BCPAP and TPC1), clonogenicity assay was performed with Y15 treatment. Y15 caused decreased colony formation in each cell line in a dose-dependent manner (Figure 5A). K1 demonstrated the least response, with over 50% colony inhibition at 8 μM Y15, which is consistent with this cell line having the highest IC_50_ value by MTS assay. BCPAP was the cell line that showed the most response with inhibition of over 50% of colonies at 3 μM Y15. TPC1 cells demonstrated a response in between, with over 50% inhibition of colonies at 5 μM Y15. The colony counts were graphed to better visualize the difference between the three cell lines (Figure 5B). Thus, Y15 caused dose-dependent decrease of colony formation in all papillary thyroid cancer cell lines.

In addition, we tested the effect of PF-04554878 inhibitor on clonogenicity in TPC1 and K1 papillary thyroid cancer cells. In both cell lines PF-04554878 decreased colony growth. Complete colony inhibition was found in 6 μM and 8 μM in TPC1 and K1, respectively (Figure 5C). Thus, PF inhibitor also effectively dose-dependently decreased colony formation in papillary thyroid cancer cell lines.

### Y15 treatment increased necrosis in papillary and medullary thyroid cancer cell lines

**Figure 6:**
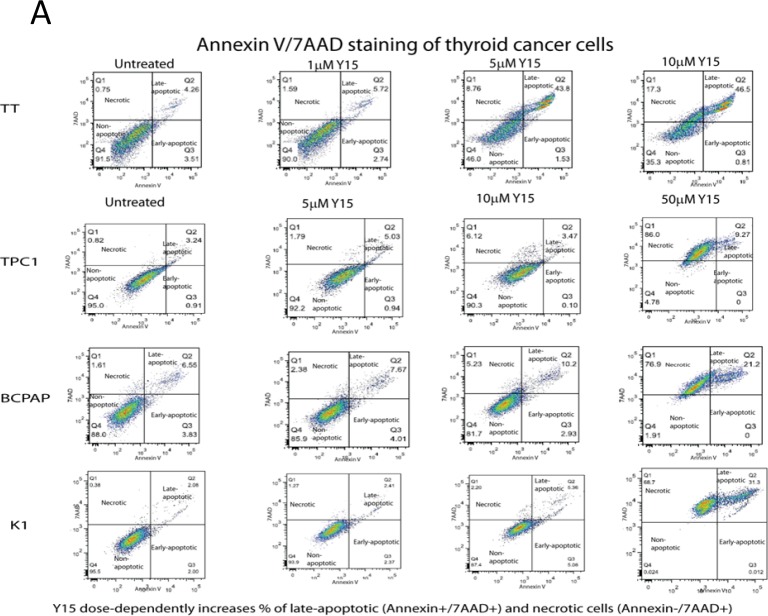

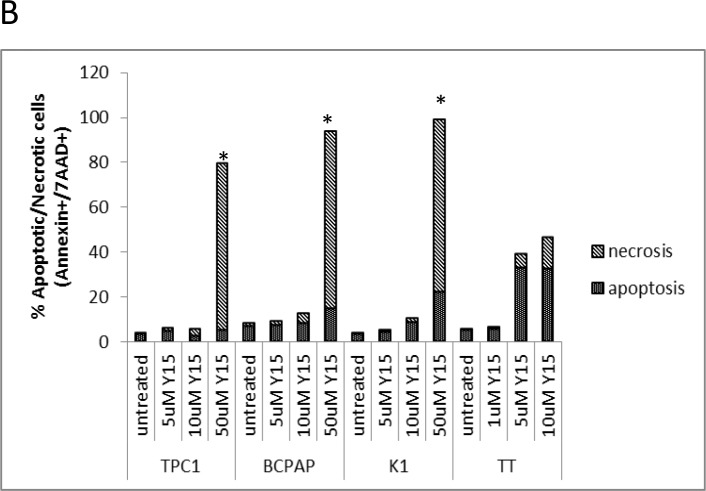
Y15 increased necrosis in a dose-dependent manner in thyroid cancer cell lines **(A)** Y15 treated cells demonstrated dose-dependent increase of necrosis detected by staining with Annexin V and Propidium iodide in four thyroid cancer cell lines. **(B)** Y15 increased necrosis in a dose-dependent manner in thyroid cancer cell lines, with statistical significance found at 50 μM doses in TPC1, BCPAP, and K1. *p<0.05 by Student's t-test.

Next, we analyzed cell death effects of Y15 in thyroid cancer cell lines by Annexin staining. Cells were treated with increasing doses of Y15 and apoptosis assay was performed with Annexin V/7AAD staining by flow cytometry. The necrosis increased in a dose dependent manner. As demonstrated previously in the MTS assay, the medullary cell line, TT, was most sensitive to Y15 treatment as seen in the lower doses of Y15 that were used. These results demonstrated an increase of cell death even at the lowest dose of Y15 (5 μM), inducing cell death in 45.33% of TT cells (Figure 6A). Conversely, the three papillary cell lines, TPC1, BCPAP, and K1, when treated with higher concentrations of Y15 induced statistically significant, p <0.05, necrosis at 50 μM 86%, 76.9%, and 68.7% necrosis, respectively compared to minimal levels in untreated control cells (0.82%, 1.61%, and 0.38%) (Figure 6B). Thus, Y15 caused a dose-dependent increase in cell death in all thyroid cell lines.

### Papillary thyroid cancer cell lines have common and unique gene changes in response to Y15

**Figure 7:**
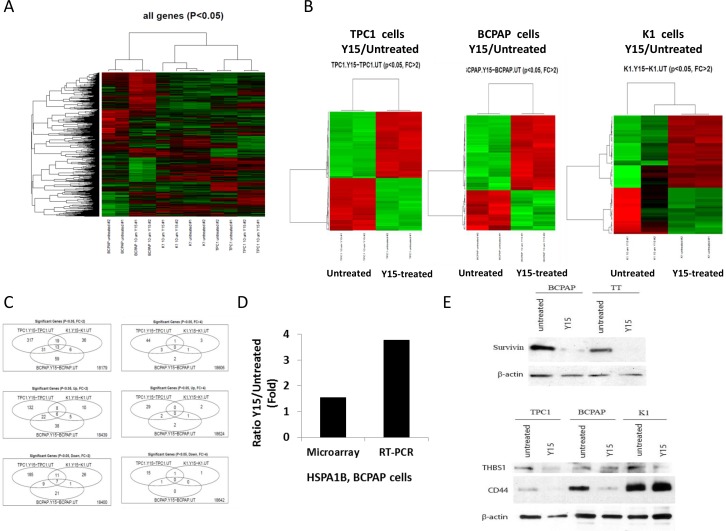
Y15 induced significant gene changes in papillary thyroid cancer cell lines **(A)** Heat map of gene changes among Y15-treated and untreated TPC1, BCPAP and K1 cell samples in duplicates. **(B)** Heat maps comparing Y15 treated to untreated cells in TPC1, K1, and BCPAP cell lines showed statistically significant gene changes over two-fold with Y15 treatment. In each heat map the green represents the gene up-regulation and the red represents the gene down-regulation. **(C)** Venn diagrams demonstrate the number of common and unique genes significantly changed (up and down-regulated) over two-fold and over four-fold with Y15 treatment in TPC1, K1, and BCPAP cell lines. **(D)** HSPA1B gene increase with Y15 by microarray and qRT-PCR analysis in BCPAP cells. qRT-PCR confirmed up-regulated gene detected by microarray analysis. **(E)** Western blotting confirmed gene expression changes with Y15 treatment detected with microarray. Upper panel shows decreased expression of survivin in BCPAP and TT cells with Y15 treatment with beta-actin used as a control. Lower panels show decreased expression of THBS1 in TPC1, BCPAP, and K1 cells. Lower panel shows decreased expression of CD44 in TPC1 and BCPAP cells and constant expression in resistant K1 cells validating microarray data.

In order to detect and compare gene changes in response to Y15 treatment between cell lines, microarray assay was performed in untreated and Y15 treated papillary thyroid cell lines. The heat map of significant gene changes by Y15 in TPC1, BCPAP and K1 cell lines are shown in Figure 7A (Figure 7A). Distinct gene changes over two-fold were found in TPC1, BCPAP, and K1 cell lines with Y15 treatment as seen in the heat maps (Figure 7B). The overlap between genes changed was relatively small with only 13 genes changed over two-fold that were changed in TPC1, BCPAP and K1. The gene expression changes in response to Y15 common among TPC1, BCPAP, and K1 cell lines are shown in Table S1. The unique genes significantly changed in Y15 treated TPC1 cells are listed in Table S2. Similarly, genes significantly changed in Y15-treated K1 and BCPAP cells are listed in Table S3 and Table S4. Conversely there were 317, 59, and 36 specific genes changed over two-fold in each TPC1, BCPAP, and K1 respectively. There were significantly less genes changed over four-fold in these treatment groups with 44, 2, and 3 genes changed in each TPC1, BCPAP, and K1 respectively and 5 genes shared among two out of three cell lines (Figure 7C). To validate the gene changes detected in the microarray study, RT-PCR (Figure 7D) and Western blotting (Figure 7E) was used with several genes. The increased heat-shock HSPA1B gene expression was detected by microarray and RT-PCR. HSPA1B was found to change 3.7 fold with Y15 treatment by RT-PCR validating the 1.5 fold increase by microarray analysis (Figure 7D). The decreased expression of BIRC5, encoding survivin, in TT and BCPAP cells with Y15 was found by microarray and was validated by Western blotting (Figure 7E). Decreased expression of THBS1 and CD44, a cancer stem cell marker, in TPC1 and BCPAP cells with Y15 that were detected by microarray were also validated by Western blotting (Figure 7E, left and middle panels). Additionally unchanged or not significantly changed expression of THBS1 and CD44 expression with Y15 in resistant K1 cells were found by microarray and were also detected and validated with Western blot (Figure 7E, right panels). Thus, we validated gene expression levels and detected common and unique gene changes in all three cell lines in response to Y15 (Supplemental Tables 1-4).

### Y15 induced gene changes distinct from those induced by PF-04554878

**Figure 8:**
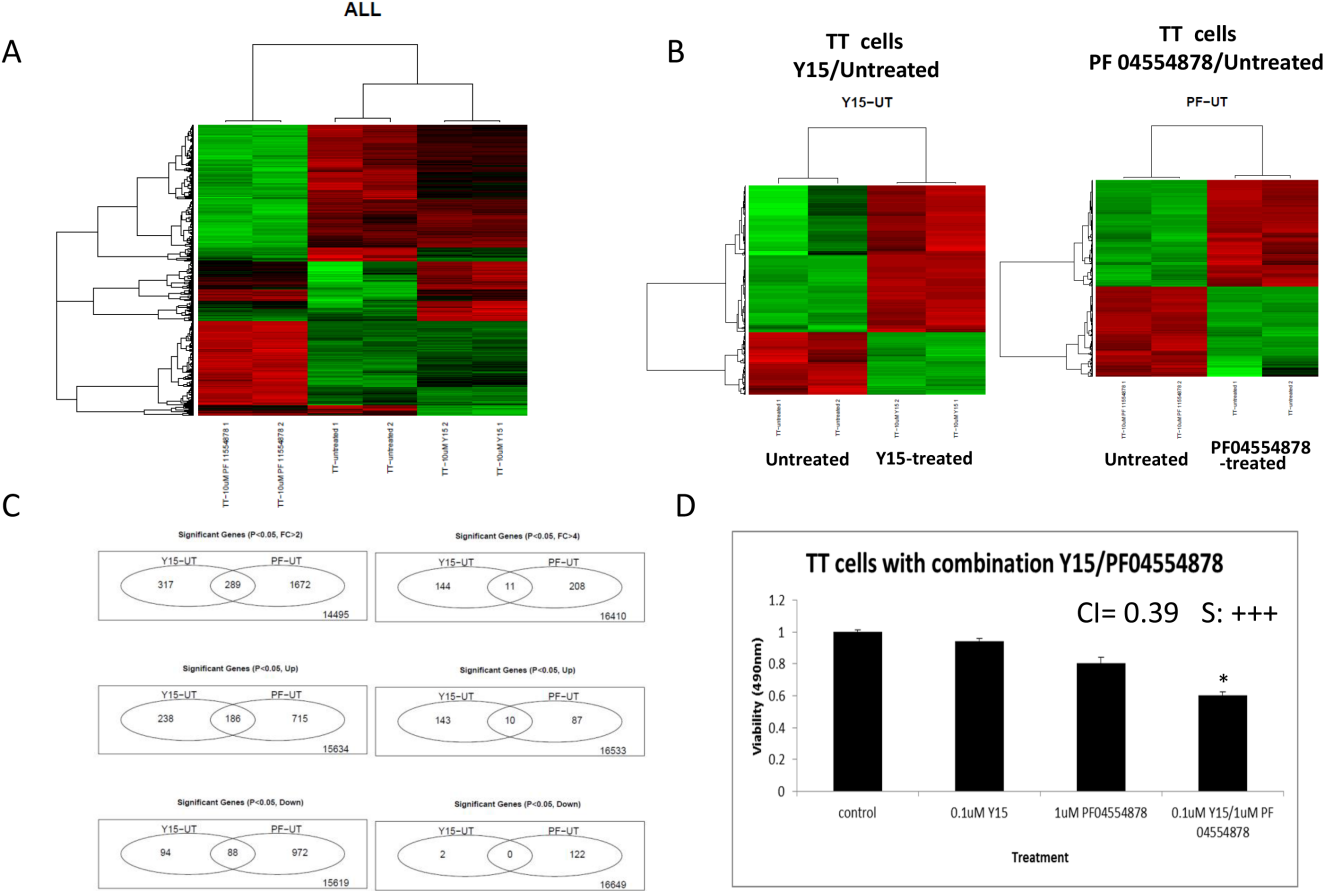
Y15 and PF-04554878 induced significant gene changes in medullary thyroid cancer TT cells **(A)** Heat map with all treatment groups demonstrated consistent gene changes between duplicates in treatment groups. The green represents gene up-regulation and the red represents gene down-regulation. **(B)** Heat maps comparing Y15 treated and PF-04554878 treated to untreated TT cells. **(C)** Venn diagrams demonstrate the number of common and unique genes significantly changed over two-fold and over four-fold, both up and down, in TT cells by Y15 and PF-04554878 treatment. **(D)** MTS assay demonstrated synergism with combination 0.1 μM Y15 and 1 μM PF-04554878 in TT cells. The combination index (CI) for these doses is equal to 0.39. Combination index for each was calculated using CompuSyn. *p<0.05 by Student's t-test.

Since Y15 and PF-04554878 had similar sensitivities in TT cells by MTT assay, but different levels of Y397FAK and FAK expression in response to these inhibitors (Figure 2E), we used microarray analysis to analyze the differences in gene expression in response to two inhibitors. We performed microarray gene expression profiling study in TT cells treated with Y15 and PF-04554878, as well as untreated cells, and compared the gene expression profiles. Gene changes within each treatment group overall are shown in a heat map (Figure 8A). Overall gene changes are also shown between untreated cells and Y15 treated cells and PF-04554878 cells (Figure 8B). The common genes changed with Y15 and PF-04554878 treatment are shown in Table S5. Y15 caused 144 genes to change over 4 fold and PF-04554878 caused 208 gene changes over 4 fold (p<0.05). Among all those genes changed to that degree, only 11 were shared between the two treatments (Figure 8C). Common genes changed include those involved in apoptosis, cell cycle, mitosis, and migration. Examples of these genes changed with both Y15 and PF-04554878 treatment include up-regulated CCDC125 and AEN and down-regulated CCNA2, KIF11, KIF20A, and KIF4A. The unique up-regulated genes in Y15-treated TT cells include genes involved in apoptosis, heat shock, and transcription: GADD45A, DDIT3, HSPA1B, and JUN. Genes down-regulated with Y15 include genes with functions including adhesion, cell division, transcription, and anti-apoptosis: CD9, TACC1, KIF23, POLR2B, and BIRC5. The unique genes up and down-regulated by Y15 in TT cells are listed in Table S6. PF-04554878 caused significant up-regulation of survival genes, transcription regulators, and oncogenes, such as ERBB3, IL17B, RASL11B, and RAF1. Those genes down-regulated by PF-04554878 include heat shock, apoptosis and anti-migration; examples include HSPA8, DIABLO, BIK, and ARHGDIA. The unique significant genes up and down-regulated by PF-04554878 in TT cells are shown in Table S7. Thus, these data revealed specific and common gene expression pathways in response to different types of FAK inhibitors: Y15 and PF-04554878.

### TT cells Y15 demonstrated synergism with PF-04554878 in medullary thyroid TT cells

Since we detected different gene expression changes in response to Y15 and PF-04554878 inhibitor in TT cells, we tested the combination of Y15 and PF-04554878 for synergistic effect (Figure 8D). PF-04554878 in combination with Y15 demonstrated synergy (combination index of 0.39), with very low doses of 0.1 μM Y15 and 1 μM PF-04554878 (Figure 8D). This data clearly show that Y15 and PF-04554878 synergize and is consistent with gene expression differences detected by microarray analysis in Y15 and PF-04554878-treated TT cells.

### Y15 demonstrated synergism in resistant thyroid K1 cells with Cabozantinib, Sorafenib, Pazopanib, and Sunitinib

**Figure 9:**
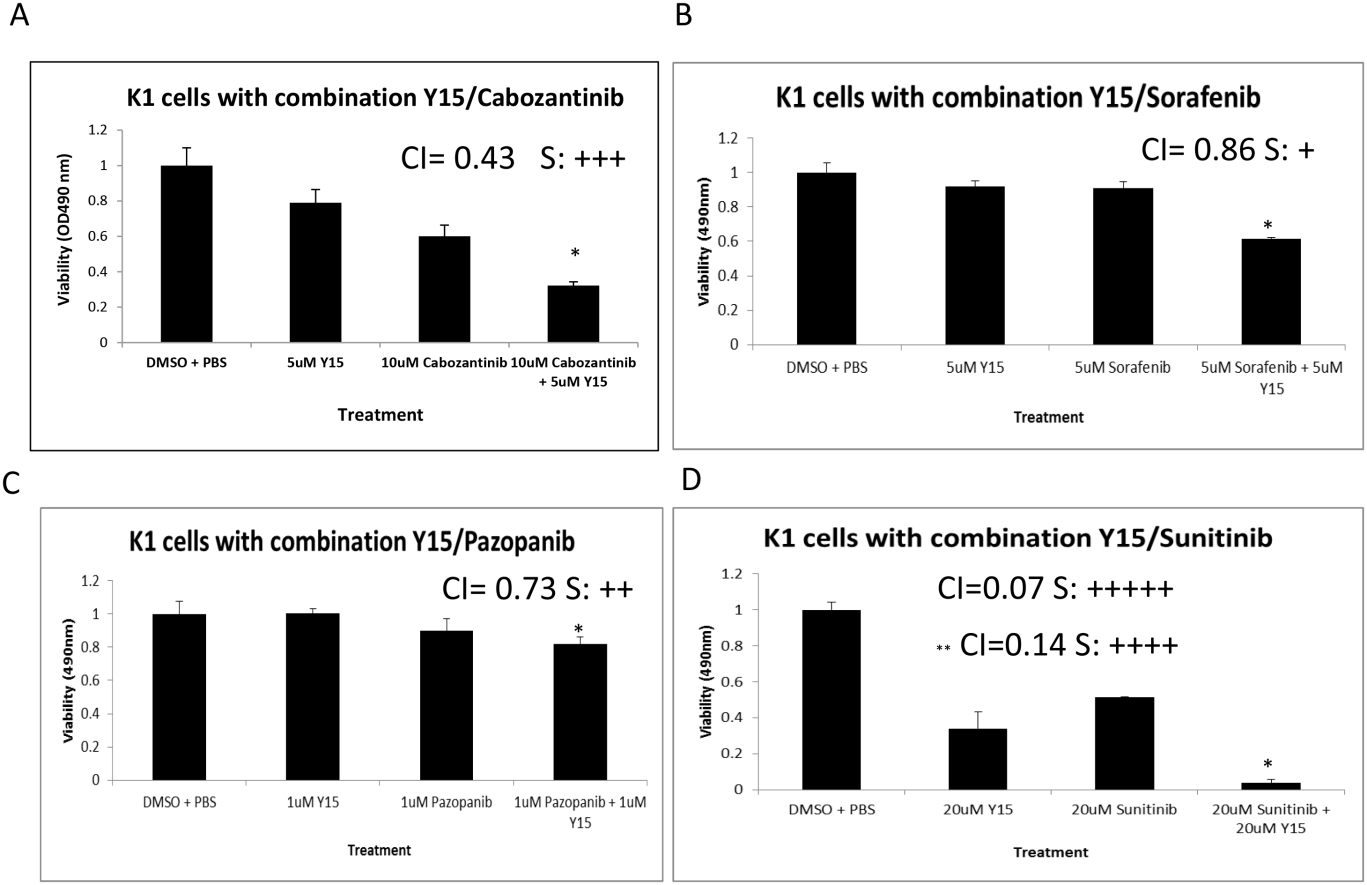
Y15 demonstrates synergism in decreasing cell viability with Cabozantinib, Sorafenib, Pazopanib, and Sunitinib in K1 resistant thyroid cells **(A)** MTS assay demonstrated synergism with combination 5 μM Y15 and 10 μM Cabozantinib in K1 cells. The combination index is equal to 0.43. **(B)** MTS assay demonstrated slight synergism with combination 5 μM Y15 and 5 μM Sorafenib in K1 cells. The combination index is equal to 0.86 with 5 μM of Y15 and Sorafenib. **(C)** MTS assay demonstrated moderate synergism with combination 1 μM Y15 and 1 μM Pazopanib in K1 cells and combination index 0.73. **(D)** MTS assay demonstrated very strong synergism with combination 20 μM Y15 and 20 μM Sunitinib in K1 cells and combination index 0.07 and with combination index 0.14 at 10 μM of Y15 and 1 μM of Sunitinib (marked by **). *p<0.05 by Student's t-test.

Since we detected that the K1 cell line was more resistant to Y15, we used combination of Y15 with other targeted therapeutics approved for clinical trials (Sorafenib, Pazopanib and Sunitinib) in this cell line [4]. To investigate the combinatorial effect of Y15 with a drug approved for clinical metastatic thyroid cancer treatment, Cabozantinib was tested with Y15 [10]. MTS assay in the most resistant K1 cells was used to evaluate the effect of these drugs both independently, with each drug alone, and in combination. We demonstrate that Y15 has synergy with Cabozantinib, Sorafenib, Pazopanib, and Sunitinib at low doses. Bliss synergism with combination index of 0.43 was detected with 5 μM Y15 and 10 μM Cabozantinib (Figure 9A). 5 μM Y15 and 5 μM Sorafenib produced synergy with combination index of 0.86 (Figure 9B). Synergism was found with very low doses of 1 μM Y15 with 1 μM Pazopanib and combination index 0.73 (Figure 9C). With slightly higher doses strong synergy was found with 20 μM Y15 with 20 μM Sunitinib; combination index was 0.07 Sunitinib (Figure 9D). At lower doses of Y15 at 10 μM and 1 μM Sunitinib also demonstrated strong synergy; combination index was 0.14 (Figure 9D). These combination indexes indicate that Y15 with Sunitinib has very strong synergism, with Pazopanib has moderate synergism, with Cabozantinib has synergism, and with Sorafenib has slight synergism.

## DISCUSSION

FAK has previously been shown to be up-regulated in many cancers, including thyroid [11]. Our lab previously showed FAK inhibition through drug Y15 decreases cell viability, clonogenicity and tumor growth in colon cancer and breast cancer cells. Due to the association of greater FAK overexpression with more aggressive thyroid tumors, FAK inhibition is a viable therapeutic strategy, especially in more aggressive phenotypes [11]. In this study we for the first time showed the role of FAK inhibitor Y15 in thyroid cancer and compared our results between cell lines. Compared with breast and colon cancer cells [8, 9], the medullary cell line was more sensitive to Y15 and combination treatment. The papillary cell lines were more resistant to Y15 than colon cancer cells, but more sensitive than breast cancer cells. This demonstrates that Y15 is effective in both sensitive and drug resistant cell lines.

In each thyroid cancer cell line FAK and Y397 phosphorylated FAK were expressed in different levels. In each cell line pY397 FAK expression decreased with Y15 treatment in a dose-dependent manner, but to different degrees, consistent with data in colon cancer [9]. The medullary cell line TT was most sensitive to pY397 FAK expression inhibition with Y15. The papillary cell line BCPAP was the least sensitive and papillary cell lines K1 and TPC1 were intermediately sensitive to Y15 treatment. Conversely, PF-04554878 did not as significantly decrease pY397 FAK expression in these cell lines, compared with Y15. This is in contrast with the previously published research on PF-04554878, in which pY397 FAK expression was significantly decreased with the drug in ovarian cancer cell lines [12]. We observed decreased AKT in response to PF-04554878 that can be explored in thyroid cancer cells in the future. Our micro-array study demonstrated several cross-linked pathways with AKT, affected by PF04554878 inhibitor. This indicates PF-04554878 is likely working through a different mechanism in thyroid cancer cell lines and this previously undescribed mechanism is causing resistance to treatment. We have demonstrated that in thyroid cancer cell lines PF-04554878 does not decrease pY397 FAK and FAK as significantly as in other cancers and this is associated with higher resistance of thyroid cancer cell lines to PF-04554878. In addition, the PF-04554878 inhibitor can work more effectively in cancer stem cells and in 3-D conditions, as was observed by other authors [12]. This can be explored in future studies in thyroid cancer cells.

The degree to which each papillary thyroid cancer cell line decreased in cell viability with Y15 treatment was correlated with the levels of pY397 FAK. The higher decreased level of Y397-FAK and FAK correlated to a higher sensitivity to Y15 treatment. Therefore, TT was the most sensitive and K1 the least, with BCPAP and TPC1 showing intermediate inhibition of cell viability with Y15. Because our small molecule inhibitor targets the Y397 phosphorylation site, it is consistent that it would cause more of an effect with a relatively higher amount of phosphorylated FAK. The medullary cell line was highly sensitive to both Y15 and PF-04554878 suggesting different signaling versus papillary cell lines in this type of thyroid cancer. Two out of the three papillary cell lines, TPC1 and BCPAP, were more sensitive to Y15 than PF-04554878; the last papillary cell line, K1, was more sensitive to PF-04554878. This indicates the two FAK inhibitors effect cancer cells through a different mechanism. Y15 was also shown to increase detachment and decrease clonogenicity in each cell line in a dose-dependent manner. PF-04554878 decreased clonogenicity in two papillary thyroid cancer cell lines to a greater degree than Y15. This can be explained by a slower short-term mechanism of action for PF-04554878 that does not show as much effect in 24 hour experiments, but appears in a two week clonogenicity assay.

The mechanism by which Y15 exerted these effects was shown to be through a combination of apoptosis and necrosis. Necrosis was detected at the highest doses, but a lower highest dose likely would have shown continued increase in apoptosis. Likely at the highest dose, the necrosis is induced as was shown in breast cancer cell line BT474 [8]. Y15 increased detachment and decrease clonogenicity through an apoptotic mechanism in colon cancer, as we found here in thyroid cancer [9]. Thus, Y15 can work through apoptosis/necrosis in thyroid cancer cells.

To investigate the mechanism of Y15 in the papillary thyroid carcinoma cell lines a microarray gene profile was obtained and compared between cell lines. There were some gene overlaps between genes changed with Y15 in the three cell lines, but none over 4-fold in all three lines. This indicates that each cell line has different protective mechanisms in response to Y15 treatment or the common responses induce small gene changes. BCPAP, TPC1, and K1 cell lines are genetically distinct cell lines from papillary thyroid tumors with varying mutations [13]. For example, BCPAP has BRAF and p53 mutations. K1 has the same BRAF mutation as BCPAP cell line, a PI3KCA mutation, and a different p53 mutation [14]. TPC1 has the RET/PTC1 rearrangement and does not share any mutations with the other papillary cell lines [14]. Different mutations can explain different significant gene changes with Y15 in these cell lines. There were genes involved in the cell cycle that were up-regulated, including ASNS and GDF15 as well as genes involved in apoptosis, including PPP1R15A and BEX2. These gene changes could explain the apoptotic mechanism of Y15. Also multiple genes involved in mitosis were down regulated including ASPM, CENPA, and CENPF, consistent with previously shown role of FAK in the nucleus [15]. Interestingly HAS3, the gene encoding hyaluronan synthase 3, was down-regulated 2.04 fold by Y15 in TPC1 cells. Previously it was shown that dual FAK and HAS3 inhibition caused synergistic inhibition of colon cancer cell viability [16]. The down-regulation of HAS3 by Y15 confirms that these work though an overlapping mechanism.

A member of the heat shock family, HSPAIB, was up-regulated 3.32 fold in TPC1 cells. Heat shock proteins are a major part of the stress response and were previously also found to be increased with Y15 treatment in colon cancer cells [16]. This indicates heat shock proteins are also involved in avoiding apoptosis in the TPC1 cell line, possibly as a mechanism of resistance to drug treatment. In the K1 cell line, numerous chemokine ligands were up-regulated with Y15 treatment. Chemokine ligands have been shown to be increased during the development of papillary thyroid tumors [17]. The K1 cell line has shown increased resistance to Y15 treatment and up-regulation of chemokine ligands is likely an indication of activating a tumorigenic response to counteract treatment. In the BCPAP cell line, many genes involved in inflammation and the immune response were up-regulated. One of these, IL1B, has been shown to increase CCL2 in follicular thyroid carcinoma, leading to lymph node metastasis [18]. Up-regulation of these genes likely indicates resistance to Y15 treatment and targeting these pathways in addition to Y15 could lead to an increased response and will be tested in subsequent studies.

To compare and contrast the mechanisms of action for Y15 and PF-04554878 we identified common and specific genes changed by both in the medullary thyroid TT cell line. Multiple kinesins were down-regulated by both drugs, including KIF11, KIF20A, and KIF 4A. Kinesins are involved in multiple cellular processes, including mitosis and intracellular transport, and drugs targeting kinesins are in clinical trials for treatment of cancer [19]. Multiple kinesins are involved in the development of cancer, including KIF11 and KIF4A, which were down-regulated by both FAK inhibitors. The kinesin pathway could be one mechanism through which PF-04554878 and Y15 interact to synergistically inhibit thyroid cancer.

Both Y15 and PF-04554878 also up-regulated encoding of AEN, apoptosis enhancing nuclease, in TT cells; Y15 by two-fold and PF-04554878 by three-fold. AEN is directly induced by p53 and subsequently acts to induce apoptosis [20]. Up-regulation of AEN by Y15 and PF-04554878 explains both the apoptotic mechanism through which Y15 works and the synergism with PF-04554878 on cell viability. Gene changes of AEN and the kinesin family could indicate the mechanism through which Y15 causes its effects on cells and through which it synergizes with PF-04554878.

Also among genes involved in apoptosis, GADD45A, GADD45B, and GADD45G, were up-regulated with Y15 in the TT cell line. These genes were up-regulated significantly 2.73 to 3.42 times, while they were not changed significantly with PF-04554878 treatment. GADD45G is similarly decreased in anaplastic thyroid cancer cells and expression of the gene induces apoptosis [20]. GADD45 proteins activate the JUN pathway as a stress response and regulate cell growth and apoptosis [21]. Our findings were consistent with this as we found JUN to also be increased with Y15 treatment 7.77 fold. This supports Y15 working through an apoptotic mechanism and control of cell death represents an effective treatment strategy.

The expression of BIRC5 gene that encodes survivin was decreased with Y15 treatment. BIRC5 was decreased 0.49-fold in Y15 treated TT cells, while it was statistically significantly increased 1.26-fold by PF-04554878. Survivin is expressed highly in thyroid cancer tumors and associated with metastasis and a worse prognosis [22]. Survivin blocks both the mitochondrial apoptosis pathway and the death receptor pathway for an anti-apoptotic effect. Again, this indicates Y15 works through multiple genes that PF-04554878 does not to induce apoptosis. These genes changes signify Y15 and PF-04554878 work through a distinct mechanism, but the similar classes of genes changed and shared genes could explain the synergy with combination of the drugs.

We also demonstrated synergy of Y15 and PF-04554878 in TT cells. PF-04554878, now known as Defactinib, is currently undergoing clinical trials in multiple cancers including mesothelioma, ovarian and lung. PF-04554878 was shown to decrease tumor volume in a chemotherapy-resistant ovarian tumor model through decreased AKT and YB-1 phosphorylation [12]. FAK is also involved in the AKT survival pathway that is down-stream of the Y397 site and this is a possible mechanism for the synergistic effect on thyroid cancer of Y15 and PF-04554878. The detected differences among Y15 and PF-04554878 inhibitors and common genes by microarray expression also can explain the synergistic effect.

In this report, for the first time, synergy was demonstrated with Y15 and Cabozantinib, Sorafenib, Pazopanib, and Sunitinib in the most resistant cell line, K1. Papillary thyroid cancer cell line K1 has a BRAF mutation, a PI3KCA mutation, and a p53 mutation [14]. The presence of a BRAF mutation confers a worse prognosis in patients, correlating our finding of resistance to Y15 [4]. These targeted drugs Cabozantinib, Sorafenib, Pazopanib, and Sunitinib all inhibit VEGFR, among other tyrosine kinases. FAK and VEGFR both interact with integrins and Src and FAK inhibition prevents vascular permeability induced by VEGFR [21], therefore it would be expected that drugs targeting VEGFR would synergize with a FAK inhibitor.

This study indicates that FAK inhibition through Y15 causes dose-dependent decreases in cell viability, clonogenicity, and attachment through an apoptotic and necrotic mechanism that synergizes with current and investigational therapeutics and this inhibition is related to the relative amount of active FAK in each cell line. The study identified common and unique pathways in response to Y15 in three cell lines and detected differences and common pathways between Y15 and PF-04554878 inhibitor. Future experiments are needed to further characterize the mechanism through which Y15 causes its apoptotic effects.

## SUPPLEMENTARY TABLES


