# Supplementary material for: FAK inhibition with small molecule inhibitor Y15 decreases viability, clonogenicity, and cell attachment in thyroid cancer cell lines and synergizes with targeted therapeutics
Source: Oncotarget. 2014 Aug 25;5(17):7945–59. doi: 10.18632/oncotarget.2381 (PMC4202172; doi:10.18632/oncotarget.2381)
Supplement: Supplementary file 1 [file oncotarget-05-7945-s001.pdf]

SUPPLEMENTARY TABLES

Supplemental Table S1. Common and significantly up-regulated and down-regulated genes (>2 fold in two lines) in Y15-treated papillary thyroid cancer cell lines TPC1, BCPAP, and K1, p<0.05

Entrez	Symbol	Name	Fold Change			Function
			TPC1	K1	BCPAP	
**Up-regulated genes**						
440	ASNS	Asparagine synthetase	4.61	2.94	4.96	Cell cycle (asparagine synthesis)
11009	IL24	Interleukin 24	3.20	2.69	2.57	Cell differentiation
9518	GDF15	Growth differentiation factor 15	3.17	7.27	6.14	Cell cycle (tissue differentiation regulator)
57761	TRIB3	Tribbles homolog 3 (Drosophila)	5.63	3.35	5.56	Transcription (blocks AKT, inhibits NF*K*B, regulates MAP kinases
4314	MMP3	Matrix metallopeptidase 3	1.12*	6.85	3.17	Breakdown of ECM (tumor initiation, wound repair, atherosclerosis, tissue remodeling)
3310	HSPA6	Heat shock protein 6	11.99	1.00*	3.30	Heat Shock
7378	UPP1	Uridine phosphorylase 1	4.37	1.74	3.02	Nucleotide synthesis
23645	PPP1R15A	Protein phosphatase 1, regulatory Subunit 15A	5.11	2.22	2.43	Apoptosis
29968	PSAT1	Phosphoserine aminotransferase 1	3.91	2.66	2.76	Amino acid biosynthesis
84707	BEX2	Brain expressed x-linked 2	3.53	1.52	3.10	Apoptosis
1026	CDKN1A	Cyclin-dependent kinase inhibitor1A	5.21	2.17	1.54	Inhibits cellular proliferation
1051	CEBPB	CCAAT/enhancer binding protein beta	3.43	1.40	2.10	Regulates acute-phase reaction
**Down-regulated genes**						
10112	KIF20A	Kinesin family member 20A	0.21	0.37	0.22	Movement of organelles
84952	CGNL1	Cingulin-like 1	0.19	0.25	0.36	Anchoring the apical junctional complex
100133941	CD24	CD24 molecule	0.19	0.53	0.44	Modulates B-cell activation responses
259266	ASPM	Asp homolog, microcephaly assoc.	0.29	0.48	0.44	Mitotic spindle regulation

Down-regulated genes						
**Entrez**	**Symbol**	**Name**	**Fold Change**			**Function**
			**TPC1**	**K1**	**BCPAP**	
7057	THBS1	Thrombospondin 1	0.21	0.93*	0.36	Cell-to-cell interactions
9133	CCNB2	Cyclin B2	0.33	0.39	0.47	Cell cycle regulation
891	CCNB1	Cyclin B1	0.44	0.57	0.38	Cell cycle regulation
1033	CDKN3	Cyclin-dependent kinase inhibitor 3	0.41	0.38	0.45	Cell cycle regulation
1058	CENPA	Centromere protein A	0.39	0.46	0.48	Mitosis
1063	CENPF	Centromere protein F	0.30	0.51	0.43	Mitosis
112464	PRKCDBP	Protein kinase C, delta binding Protein	0.33	0.47	0.47	Immune potentiation (down regulated in cancer)
991	CDC20	Cell division cycle 20 homolog	0.36	0.51	0.42	Cell cycle regulation
332	BIRC5	Baculoviral IAP repeat-containing 5	0.41	0.60*	0.51	Proliferation, Anti-apoptotic

*not significant, $p>0.05$

Supplemental Table S2. Specific, significantly up-regulated and down-regulated genes (>2 fold) in Y15-treated papillary thyroid cancer TPC1 cells, $p<0.05$

Entrez	Symbol	Title	Fold Change	Function
**Up-regulated genes**				
1026	CDKN1A	Cyclin-Dependent Kinase Inhibitor	5.21	Cell cycle
3162	HMOX1	Heme oxygenase	11.59	Heme catabolism
3576	IL8	Interleukin 8	11.48	Inflammatory response
3936	LCP1	Lymphocyte cytosolic protein 1	5.66	Actin-binding protein
3304	HSPA1B	Heat shock protein 1B	3.32	Heat shock
9188	DDX21	DEAD box polypeptide 21	2.21	Transcription
3821	KLRC1	Killer cell lectin-like receptor subfamily C, Member 1	2.88	Antigen recognition
57574	MARCH4	Membrane-associated ring finger 4	4.98	Membrane transport
390	RND3	Rho family GTPase 3	4.48	Loss of adhesion
7296	TXNRD1	Thioredoxin reductase 1	4.04	Selenium metabolism, oxidative stress protection
6447	SCG5	Secretogranin V	4.03	Regulates pituitary hormone secretion
8942	KYNU	Kynureninase	4.01	NAD cofactor synthesis
**Down-regulated genes**				
2312	FLG	Filaggrin	0.22	Epidermal development
51523	CXXC5	CXXC finger 5	0.22	Cell cycle
83543	AIF1L	Allograft inflammatory factor 1- like	0.22	Actin binding
6319	SCD	Stearoyl-CoA desaturase	0.22	Fatty acid biosynthesis
3773	KCNJ16	Potassium inwardly-rectifying channel	0.21	Potassium channel
3486	IGFBP3	Insulin-like growth factor binding protein 3	0.20	Modulates IGF
10786	SLC17A3	Solute carrier family 17, member 3	0.20	Renal tubule transporter
1052	CEBPD	CCAAT/enhancer binding protein delta	0.18	Immune response
1462	VCAN	Versican	0.17	Extracellular matrix component
29775	CARD10	Caspase recruitment domain family, Member 10	0.47	Apoptosis
1028	CDKN1C	Cyclin-dependent kinase inhibitor 1C	0.48	Down-regulates proliferation
1612	DAPK1	Death-associated protein kinase 1	0.25	Promotes apoptosis
3038	HAS3	Hyaluronan synthase 3	0.49	Hyaluronic acid synthesis
4214	MAP3K1	Mitogen-activated protein kinase kinase Kinase 1	0.49	Apoptosis
10397	NDRG1	N-myc downstream regulated gene 1	0.38	Stress response

Supplemental Table S3. Specific, significantly up-regulated and down-regulated genes (>2 fold) in Y15-treated papillary thyroid cancer K1 cells, p<0.05

Entrez	Symbol	Title	Fold Change	Function
**Up-regulated genes**				
5055	SERPINB2	Serpin peptidase inhibitor, clade B, 2	5.36	Inhibits urokinase-type plasminogen activator
6364	CCL20	Chemokine ligand 20	2.03	Immune function
6348	CCL3	Chemokine ligand 3	2.48	Inflammation
414062	CCL3L3	Chemokine ligand 3- like 3	2.84	Immune function
11082	ESM1	Endothelial cell-specific molecule 1	2.65	Angiogenesis
10135	NAMPT	Nicotinamide phosphoribosyltransferase	2.73	NAD biosynthesis
5743	PTGS2	Prostaglandin-endoperoxide synthase 2	3.63	Inflammation
10221	TRIB1	Tribbles homolog 1	3.00	Regulates activation of MAP kinases
**Down-regulated genes**				
9982	FGFBP1	Fibroblast growth factor binding protein 1	0.20	Angiogenesis
929	CD14	CD14	0.50	Immune function
32	ACACB	Acetyl-Coezyme A carboxylase beta	0.45	Fatty acid synthesis
92370	ACPL2	Acid phosphatase-like 2	0.46	Acid phosphatase pathway
80117	ARL14	ADP-ribosylation factor-like 14	0.38	Vesicle movement
10882	C1QL1	Complement component 1, q Subcomponent-like 1	0.43	Regulates excitatory synapses
1293	COL6A3	Collagen, type IV, alpha 3	0.33	Cell-binding
115908	CTHRC1	Collagen triple helix repeat containing 1	0.44	Negative regulator of collagen
129804	FBLN7	Fibulin 7	0.48	Adhesion
2706	GJB2	Gap junction protein, beta 2	0.39	Gap junction channel activity
3148	HMGB2	High-mobility group box 2	0.42	Chromatin formation
22998	LIMCH1	LIM and calponin homology domains 1	0.48	Actin binding
54674	LRRN3	Leucine rich repeat neuronal 3	0.48	Protein binding
56925	LXN	Latexin	0.49	Inflammation
58538	MPP4	Membrane protein, palmitoylated 4	0.43	Photoreceptor development
25878	MXRA5	Matrix-remodelling associated 5	0.36	
79400	NOX5	NADPH oxidase, EF-hand calcium Binding domain 5	0.37	Cell growth, apoptosis
25903	OLFML2B	Olfactomedin-like 2B	0.46	Extracellular matrix binding
5564	PRKAB1	Protein kinase, AMP-activated, beta 1	0.50	Stimulates catabolic pathways
10418	SPON1	Spondin 1	0.48	Cell adhesion

Supplemental Table S4. Specific, significantly up-regulated and down-regulated genes (>2 fold) in Y15-treated papillary thyroid cancer BCPAP cells, $p<0.05$

Entrez	Symbol	Title	Fold Change	Function
**Up-regulated genes**				
54541	DDIT4	DNA-damage-inducible transcript 4	4.44	Apoptosis
4599	MX1	Myxovirus resistance 1	5.00	Dynamin family
3553	IL1B	Interleukin 1 beta	3.12	Inflammation
3569	IL6	Interleukin 6	3.37	Inflammation
3665	IRF7	Interferon regulatory factor 7	2.45	Immune response
10379	IRF9	Interferon regulatory factor 9	2.64	Immune response
9636	ISG15	ISG15 ubiquitin-like modifier	2.55	Immune response
3914	LAMB3	Laminin beta 3	2.20	Cell attachment, migration
3934	LCN2	Lipocalin 2	2.91	Apoptosis
1410	CRYAB	Crystallin, alpha B	2.67	Chaperone-like, heat shock
**Down-regulated genes**				
23705	CADM1	Cell adhesion molecule 1	0.41	Cell adhesion, tumor suppressor in NSCLC
7184	HSP90B1	Heat shock protein 90kDa beta, member 1	0.48	Heat shock
970	CD70	CD70	0.50	T-cell activation

Supplemental Table S5. Common and significantly up-regulated and down-regulated genes (>2 fold) in Y15-treated and PF04554878-treated medullary thyroid cancer TT cells, p<0.05

Entrez	Symbol	Name	Fold Change		Function
			Y15	PF04554878	
84061	MAGT1	Magnesium transporter 1	11.70	7.40	Cell membrane Mg transport
147372	CCBE1	Collagen and Ca binding EGF domains 1	5.85	4.35	Migration, ECM remodeling
5997	RGS2	Regulator of G-protein signalling 2	4.43	0.13	Cell growth inhibition
91120	ZNF682	Zinc finger protein 682	4.86	4.98	Transcription regulation
64746	ACBD3	Acyl-CoA binding domain 3	2.15	2.33	Golgi complex maintenance
64782	AEN	Apoptosis enhancing nuclease	2.08	3.02	Apoptosis
196	AHR	Aryl Hydrocarbon receptor	4.55	2.41	Transcriptional activator
326	AIRE	Autoimmune regulator	5.34	2.70	Transcriptional regulator
26993	AKAP8L	A kinase anchor protein 8-like	2.22	3.58	DNA replication regulator
6718	AKR1D1	Aldo-keo reductase family 1, member D1	3.29	2.79	Bile acid and steroid metabolism
250	ALPP	Alkaline phosphatase, placental	6.06	2.55	Metabolism
65062	ALS2CR2	Amyotrophic lateral sclerosis 2 chromosome region 2	0.45	0.35	Metabolism
9639	ARHGEF10	Rho guanine nucleotide exchange factor 10	0.47	0.43	Myelination
339231	ARL16	ADP-ribosylation factor-like 16	4.07	2.91	GTP binding
440	ASNS	Asparagine synthetase	2.45	4.26	Asparagine synthesis
29028	ATAD2	ATPase family, AAA domain containing 2	0.27	0.23	Chaperone-like
1386	ATF2	Activating transcription factor 2	2.26	2.00	Transcriptional activator
6790	AURKA	Aurora kinase A	0.37	0.12	Cell cycle regulator
9212	AURKB	Aurora kinase B	0.39	0.15	Cell cycle regulator
8553	BHLHB2	Basic helix-loop-helix domain, class B, 2	3.97	2.24	Cell differentiation
8548	BLZF1	Basic leucine zipper nuclear factor 1	4.89	2.35	Protein transport
202243	CCDC125	Coiled-coil domain containing 125	4.95	2.32	Cell migration
54908	CCDC99	Coiled-coil domain containing 99	0.37	0.28	Mitosis
890	CCNA2	Cylin A2	0.43	0.23	Cell cycle
3832	KIF11	Kinesin family member 11	0.38	0.33	Mitosis
10112	KIF20A	Kinesin family member 20A	0.33	0.29	Mitosis
24137	KIF4A	Kinesin family member 4A	0.30	0.41	Mitosis
9735	KNTC1	Kinetochore associated 1	0.43	0.39	Mitosis
286826	LIN9	Lin-9 homolog	0.47	0.47	Tumor suppressor

Supplemental Table S6. Specific and significantly up-regulated and down-regulated genes (>2 fold) in Y15-treated medullary thyroid cancer TT cells, p<0.05.

Up-regulated genes					
**Entrez**	**Symbol**	**Name**	**Fold Change**		**Function**
			**Y15**	**PF04554878**	
3310	HSPA6	Heat shock protein 6	56.92	1.05	Heat shock
3311	HSPA7	Heat shock protein 7	21.61	1.18*	Heat shock
23645	PPP1R15A	Protein phosphatase 1, regulatory subunit 15	10.02	1.47*	Apoptosis
1649	DDIT3	DNA-damage-inducible transcript 3	9.05	0.61*	Apoptosis
54541	DDIT4	DNA-damage-inducible transcript 4	6.49	1.89*	Apoptosis
3304	HSPA1B	Heat shock protein 1B	8.87	0.67	Heat shock
3303	HSPA1A	Heat shock protein 1A	7.50	0.39*	Heat shock
3725	JUN	Jun oncogene	7.77	0.73*	Transcription regulator
1410	CRYAB	Crystallin, alpha B	4.47	1.08	Chaperone-like
3337	DNAJB1	DnaJ homolog, subfamily B, member 1	6.21	0.35*	Heat shock
4189	DNAJB9	DnaJ homolog, subfamily B, member 9	5.22	0.50*	Heat shock
1843	DUSP1	Dual specificity phosphatase 1	5.46	0.71*	Inhibits proliferation
23710	GABARAPL1	GABA receptor-associated protein like 1	4.08	0.86	Autophagosome formation
9518	GDF15	Growth differentiation factor 15	5.35	1.07	Regulates tissue differentiation
51278	IER5	Immediate early response 5	5.34	0.89	Mediates cell division
84515	MCM8	Minichromosome maintenance complex 8	4.18	1.86*	DNA replication
4792	NFKBIA	Nuclear factor of kappa light polypeptide gene enhancer in B-cells inhibitor alpha	4.51	1.38*	Inflammatory response
266743	NPAS4	Neuronal PAS domain protein 4	5.25	1.16*	Transcriptional activator
26471	NUPR1	Nuclear protein transcriptional regulator 1	5.16	1.72*	Stress response
440503	PLIN5	Perilipin 5	4.37	1.75*	Protects lipid storage droplets
27314	RAB30	RAS oncogene family member	4.63	1.00	Intracellular membrane trafficking
871	SERPINKH1	Serpin peptidase inhibitor, clade H, 1	4.86	0.46*	Collagen synthesis
29950	SERTAD1	SERTA domain containing 1	4.51	0.88	Promotes cell proliferation, transcription

Up-regulated genes					
Entrez	Symbol	Name	Fold Change		Function
			Y15	PF04554878	
387700	SLC16A12	Solute carrier family 16, member 12	4.36	1.80*	Monocarboxylic acid transporter
7779	SLC30A1	Solute carrier family 30, member 1	5.37	1.43*	Zinc transport
330	BIRC3	Baculoviral IAP repeat-containing 3	3.43	1.79*	Apoptosis inhibitor
2309	FOXO3	Forkhead box O3	2.39	1.38*	Apoptosis
1647	GADD45A	Growth arrest & DNA-damage-inducible α	2.73	1.04	Apoptosis
4616	GADD45B	Growth arrest & DNA-damage-inducible β	3.34	1.11	Apoptosis
10912	GADD45G	Growth arrest & DNA-damage-inducible γ	3.42	1.12	Apoptosis
**Down-regulated genes**					
52	ACP1	Acid phosphatase 1	0.48	0.75*	Tyrosine phosphatase
115	ADCY9	Adenylate cylase 9	0.39	1.25	cAMP production
332	BIRC5	Baculoviral IAP repeat-containing 5	0.49	1.26*	Proliferation, Anti-apoptotic
23705	CADM1	Cell adhesion molecule 1	0.43	0.67*	Adhesion
4176	CD46	CD46	0.47	1.78*	Complement regulator
928	CD9	CD9	0.36	0.82	Adhesion, motility, metastasis
64105	CENPK	Centromere protein K	0.38	0.54*	Mitosis
26586	CKAP2	Cytoskeleton associated protein 2	0.46	0.79*	Cell cycle regulator
10143	CLEC3A	C-type lectin domain family 3, member A	0.48	0.83	Adhesion
1832	DSP	Desmoplakin	0.49	0.84	Adhesion
4072	EPCAM	Epithelial cell adhesion molecule	0.47	0.75*	Adhesion
2108	ETFA	Electron-transfer-flavoprotein	0.49	0.85	ATP synthesis
2194	FASN	Fatty acid synthase	0.27	0.75	Fatty acid synthesis
8836	GGH	Gamma-glutamyl hydrolase	0.42	0.98	Folic acid metabolism
10149	GPR64	G protein-coupled receptor 64	0.46	1.20*	Epididymal function
25831	HECTD1	HECT domain containing 1	0.45	0.85	Ubiquitin transport
6596	HLTF	Helicase-like transcription factor	0.49	0.68*	Transcription regulator
51155	HN1	Hematological and neurological expressed 1	0.50	1.28*	Apoptosis

(*Continued*)

Down-regulated genes					
Entrez	Symbol	Name	Fold Change		Function
			Y15	PF04554878	
3428	IFI16	Interferon, gamma-inducible protein 16	0.48	0.67*	Transcription regulator
3482	IGF2R	Insulin-like growth factor 2 receptor	0.50	1.26	Lysosomal enzyme transport
3685	ITGAV	Integrin, alpha V	0.47	1.51*	Adhesion, Migration
9928	KIF14	Kinesin family member 14	0.45	0.74*	Cell division, intracellular transport
9585	KIF20B	Kinesin family member 20B	0.35	0.57*	Cell division
9493	KIF23	Kinesin family member 23	0.49	1.21*	Cell division, intracellular transport
3838	KPNA2	Karyopherin alpha 2	0.49	0.67*	Nuclear protein import
22859	LPHN1	Latrophilin 1	0.49	1.55*	Cell adhesion, signal transduction
4628	MYH10	Myosin, heavy chain 10	0.44	0.83	Cell division, motility
4676	NAP1L4	Nucleosome assembly protein 1-like 4	0.46	0.73*	Histone chaperone
4784	NFIX	Nuclear factor I/X	0.43	0.76*	Transcription activator
5122	PCSK1	Proprotein convertase subtilisin/kexin type 1	0.45	0.69*	GI hormone metabolism
5223	PGAM1	Phosphoglycerate mutase 1	0.50	1.09	Glycolysis
5431	POLR2B	Polymerase II polypeptide B	0.47	1.18	DNA transcription
9055	PRC1	Protein regulator of cytokinesis 1	0.39	1.27*	Cell division
10935	PRDX3	Peroxiredoxin 3	0.36	1.05	Antioxidant
10549	PRDX4	Peroxiredoxin 4	0.50	0.84	Antioxidant
5631	PRPS1	Phosphoribosyl pyrophosphate synthetase 1	0.49	0.64*	Nucleotide synthesis
5902	RANBP1	RAN binding protein 1	0.47	0.70*	Cell cycle regulation
5934	RBL2	Retinoblastoma-like 2	0.48	1.20*	Cell division regulator
157506	RDH10	Retinol dehydrogenase 10	0.49	0.64*	Retinoic acid synthesis
6241	RRM2	Ribonucleotide reductase M2 polypeptide	0.47	0.68*	DNA synthesis, wnt inhibitor
5274	SERPINI1	Serpin peptidase inhibitor, clade 1, member 1	0.40	1.10	Plasmin signaling
23213	SULF1	Sulfatase 1	0.48	0.67*	Apoptosis
6867	TACC1	Transforming, acidic coiled-coil containing protein 1	0.49	0.68*	Cell division promoter, breast ca gene

Down-regulated genes					
**Entrez**	**Symbol**	**Name**	**Fold Change**		**Function**
			**Y15**	**PF04554878**	
7027	TFDP1	Transcription factor Dp-1	0.46	0.71*	DNA transcription
23505	TMEM131	Transmembrane protein 131	0.47	1.88*	Immune response
10330	TMEM4	Transmembrane protein 4	0.50	0.82*	Regulates neurite outgrowth
64759	TNS3	Tensin 3	0.50	0.60*	Migration
9189	ZBED1	Zinc finger, BED-type containing 1	0.49	0.84*	Stimulates transcription, regulates cell proliferation
80110	ZNF614	Zinc finger protein 614	0.49	1.87*	Transcription regulator

*also significant in PF04554878 treated cells, $p<0.05$

Supplemental Table S7. Specific and significantly up-regulated and down-regulated genes (>2 fold) in PF04554878-treated medullary thyroid cancer TT cells, p<0.05.

Up-regulated genes

Entrez	Symbol	Name	Fold Change		Function
			PF04554878	Y15	
10673	TNFSF13B	Tumor necrosis factor superfamily, 13b	8.90	1.24*	Proliferation, B cell activator
26959	HBP1	HMG-box transcription factor 1	8.38	2.49*	Transcriptional repressor
353376	TICAM2	Toll-like receptor adaptor molecule 2	8.06	1.02	Immune response
11083	DIDO1	Death inducer-obliterator 1	7.89	0.96	Apoptosis
57693	ZNF317	Zinc finger protein 317	7.60	2.35*	Transcription factor
1994	ELAVL1	ELAV-like 1	7.47	1.07	Anti-proliferative
22863	KIAA0831	KIAA0831	7.29	2.20*	Autophagy
8493	PPM1D	Protein phosphatase 1D magnesium-dependent, delta	7.27	1.78*	Anti-apoptosis
4084	MXD1	MAX dimerization protein 1	6.98	2.30*	Transcription repressor
27190	IL17B	Interleukin 17B	2.77	1.06	Survival, growth, differentiation
23765	IL17RA	Interleukin 17 receptor A	2.26	0.95	Survival, growth, differentiation
3609	ILF3	Interleukin enhancer binding factor 3	2.47	0.95	Transcription regulator
80895	ILKAP	Integrin-linked kinase-associated serine/Threonine phosphatase 2C	2.46	1.31*	Cell cycle regulator
6885	MAP3K7	Mitogen-activated protein kinase 7	2.82	1.03	Apoptosis
2065	ERBB3	V-erb-b2 erythroblastic leukemia viral Oncogene homolog 3 (HER3)	2.65	1.05	HER3
10520	ZNF211	Zinc finger protein 211	6.02	1.57*	Transcription regulator
4335	MNT	MAX binding protein	2.55	1.32*	Transcription repressor
58	ACTA1	Actin, alpha 1, skeletal muscle	5.87	1.04	Motility
64577	ALDH8A1	Aldehyde dehydrogenase 8 family, Member A1	2.28	1.02	Retinoic acid synthesis
9774	BCLAF1	BCL2-associated transcription factor 1	2.72	1.31*	Death-promoting transcriptional repressor
5894	RAF1	v-raf-1 murine leukemia viral oncogene Homolog 1	3.40	1.19	Oncogene
387496	RASL11A	RAS-like, family 11, member A	2.16	1.13*	Transcription regulator
8428	STK24	Serine/threonine kinase 24	2.04	1.06	Apoptosis promoter
140901	STK35	Serine/threonine kinase 35	2.43	1.30*	Regulator of actin stress fibers
221830	TWISTNB	TWIST neighbor	2.44	1.29*	DNA transcription
10628	TXNIP	Thioredoxin interacting protein	2.29	0.99	Tumor suppressor

Entrez	Symbol	Name	Fold Change		Function
			**Up-regulated genes**		
			PF04554878	Y15	
972	CD74	CD74	2.20	1.07	Regulates antigen presentation
155435	RBM33	RNA binding motif protein 33	14.48	1.53*	
375133	PI4KAP2	Phosphatidylinositol 4-kinase, catalytic, alpha polypeptide pseudogene 2	2.18	0.81	
			**Down-regulated genes**		
79191	IRX3	Iroquois homeobox 3	0.16	0.86	Neural development
55540	IL17RB	Interleukin 17 receptor B	0.47	0.70*	Survival, growth, differentiation
5685	PSMA4	Proteasome prosome macropain subunit α4	0.14	0.93	MHC peptide processing
10058	ABCB6	ATP-binding cassette, sub-family B Member 6	0.47	0.83	Heme synthesis
396	ARHGDIA	Rho GDP dissociation inhibitor alpha	0.47	0.83	Inhibits cell migration and invasion
23786	BCL2L13	BCL-like 13	0.46	0.90	Promotes apoptosis
599	BCL2L2	BCL-like 2	0.47	1.11	Promotes cell survival
100133941	CD24	CD24	0.48	1.08	Stem cell marker
8487	SIP1	Survival of motor neuron protein Interacting protein 1	0.31	0.61*	mRNA splicing
56848	SPHK2	Sphingosine kinase 2	0.40	0.95	promotes angiogenesis and tumorigenesis
7913	DEK	DEK oncogene	0.27	0.55*	chromatin binding
9935	MAFB	v-maf musculoaponeurotic fibrosarcoma Oncogene homolog B	0.46	1.16*	Hematopoiesis, oncogene/ tumor suppressor
57111	RAB25	RAB25, member RAS oncogene family	0.48	1.18*	Promotes invasion
57799	RAB40C	RAB40C, member RAS oncogene family	0.37	1.23	RAS family
286319	TUSC1	Tumor suppressor candidate 2	0.48	0.72*	Carcinogenesis
1620	DBC1	Deleted in bladder cancer 1	0.36	1.03	Transcription
9238	TBRG4	Transforming growth factor beta regulator 4	0.44	0.96	Cell cycle
9220	TIAF1	TGFB1-induced anti-apoptotic factor 1	0.49	1.08	Anti-apoptotic
638	BIK	BCL2-interacting killer	0.37	0.89	Induces apoptosis
8738	CRADD	CASP2 and RIPK1 domain containing Adaptor with death domain	0.33	0.66*	Induces apoptosis

(*Continued*)

Down-regulated genes					
**Entrez**	**Symbol**	**Name**	**Fold Change**		**Function**
			**PF04554878**	**Y15**	
9093	DNAJA3	DnaJ(Hsp40)homolog, subfamily A, mem.3	0.42	0.84*	Heat shock
1434	CSE1L	CSE1 chromosome segregation 1-like	0.27	0.70*	Nuclear protein transport
56616	DIABLO	Diablo homolog	0.13	0.42*	Promotes apoptosis
3336	HSPE1	Heat shock protein 1	0.27	0.63*	Heat shock
3312	HSPA8	Heat shock protein 8	0.26	1.05	Heat shock
2956	MSH6	MutS homolog 6	0.45	1.04	DNA mismatch repair

*also significant in Y15-treated cells, $p<0.05$
